# Efficient genetic transformation and CRISPR/Cas9‐mediated genome editing in *Lemna aequinoctialis*

**DOI:** 10.1111/pbi.13128

**Published:** 2019-05-03

**Authors:** Yu Liu, Yu Wang, Shuqing Xu, Xianfeng Tang, Jinshan Zhao, Changjiang Yu, Guo He, Hua Xu, Shumin Wang, Yali Tang, Chunxiang Fu, Yubin Ma, Gongke Zhou

**Affiliations:** ^1^ College of Resources and Environment Qingdao Agricultural University Qingdao China; ^2^ Key Laboratory of Biofuels Qingdao Engineering Research Center of Biomass Resources and Environment Shandong Provincial Key Laboratory of Energy Genetics Qingdao Institute of Bioenergy and Bioprocess Technology Chinese Academy of Sciences Qingdao China; ^3^ Institute for Evolution and Biodiversity University of Münster Münster Germany

**Keywords:** *Agrobacterium*‐mediated transformation, CRISPR/Cas9, duckweed, genome editing

## Abstract

The fast growth, ease of metabolic labelling and potential for feedstock and biofuels production make duckweeds not only an attractive model system for understanding plant biology, but also a potential future crop. However, current duckweed research is constrained by the lack of efficient genetic manipulation tools. Here, we report a case study on genome editing in a duckweed species, *Lemna aequinoctialis*, using a fast and efficient transformation and CRISPR/Cas9 tool. By optimizing currently available transformation protocols, we reduced the duration time of *Agrobacterium‐*mediated transformation to 5–6 weeks with a success rate of over 94%. Based on the optimized transformation protocol, we generated 15 (14.3% success rate) biallelic *LaPDS* mutants that showed albino phenotype using a CRISPR/Cas9 system. Investigations on CRISPR/Cas9‐mediated mutation spectrum among mutated *L. aequinoctialis* showed that most of mutations were short insertions and deletions. This study presents the first example of CRISPR/Cas9‐mediated genome editing in duckweeds, which will open new research avenues in using duckweeds for both basic and applied research.

## Introduction

Duckweeds are small, fast‐growing and morphologically simple flowering aquatic plants (Landolt, 1986; Ziegler *et al*., 2015). For decades, these features made duckweeds an attractive model for understanding fundamental biological processes of plants, for example, photoperiodic control of flowering (Cleland and Tanaka, 1979), cellular circadian clocks (Muranada and Oyama, 2016), as well as biosynthesis of auxin (Rapparini *et al*., 1999) and assimilation of sulphur (Datko *et al*., 1978). Recent studies showed that duckweeds have an efficient photosynthesis capacity, high starch accumulation rate and low lignin content, which make duckweeds excellent candidates for biofuels production (Xu *et al*., 2011, 2012). In addition, duckweeds are capable of rapidly accumulating heavy metals, nutrients and pollutants, which can be used for bio‐remediation of wastewater (Cheng and Stomp, 2009; Khellaf and Zerdaoui, 2010; Megateli *et al*., 2009; Stout *et al*., 2010; Xu *et al*., 2018). The extremely fast population doubling rate (3–5 days per asexual doubling under ideal conditions) also makes duckweeds a good candidate for the production of recombinant proteins including antigens, monoclonal antibodies and exogenous enzymes (Cox *et al*., 2006; Firsov *et al*., 2015, 2018; Khvatkov *et al*., 2018; Ko *et al*., 2011; Naik *et al*., 2012; Stomp, 2005; Sun *et al*., 2007), as well as dietary supplements for humans and animals because of its high nutritional value (Appenroth *et al*., 2017; Beukelaar *et al*., 2019; Bhanthumnavin and Mcgarry, 1971). Despite the potential for both basic research and industrial applications that established duckweeds as one of the most promising alternative future crops (Appenroth *et al*., 2015; Lam *et al*., 2014), current research and development in duckweeds are constrained by the lack of efficient genetic manipulation tools that allow to knockout or knock‐in a gene of interest in the duckweed genome within a short time.

The development of genetic manipulation tools requires both an efficient transformation protocol and an effective genome editing system. Among the 37 duckweed species that belong to five genera: *Spirodela*,* Landoltia*,* Lemna*,* Wolffiella* and *Wolffia* (Landolt, 1986; Sree *et al*., 2016), the *Agrobacterium‐*mediated transformation system has been developed in at least seven of them, including *Lemna gibba* (Cantó‐Pastor *et al*., 2015; Yamamoto *et al*., 2001), *Lemna minor* (Chhabra *et al*., 2011; Firsov *et al*., 2015, 2018; Ko *et al*., 2011; Sun *et al*., 2007; Yamamoto *et al*., 2001; Yang *et al*., 2013, 2018a), *Lemna turionifera* (Yang *et al*., 2017), *Landoltia punctata* (formerly *Spirodela oligorrhiza*) (Rival *et al*., 2008; Vunsh *et al*.,^2007^), *Spirodela polyrhiza* (Yang *et al*., 2018b), *Wolffia arrhiza* (Khvatkov *et al*., 2015, 2018) and *Wolffia globosa* (Heenatigala *et al*., 2018). However, the efficiency of the transformation varied dramatically among species (from 0.14% to 82.5%).

While using the established transformation system, one can manipulate the transcript abundance of the gene of interest through RNA interference (RNAi) or over‐expression, it can suffer from low efficiency and instability due to DNA methylation of the transgene and incomplete target gene silencing (Klose and Bird, 2006; Weinhold *et al*., 2013). Therefore, to understand the function of genes and traits in duckweeds, developing tools that can stably and precisely manipulate the gene of interest is critical. To this end, the recently developed CRIPSR/Cas9 system, which was adapted from a naturally occurring genome editing system in bacteria and has been widely used in both animals and plants, is ideal (Cao *et al*., 2016; Ma *et al*., 2016; Yang, 2017). However, to our knowledge, no study has reported using CRISPR/Cas9 in duckweeds. Due to the feature of asexual budding, which is different from many other plants on which CRISPR/Cas9 has been applied, whether and to what extent this system will work in duckweeds remains unknown.

*Lemna aequinoctialis* is one of the most widely distributed duckweed species in China (Tang *et al*., 2015; Xu *et al*., 2015). *L. aequinoctialis* has been used to study starch accumulation (Ma *et al*., 2018; Yin *et al*., 2015; Yu *et al*., 2017), waste water treatment (Yu *et al*., 2014; Zhou *et al*., 2018), endophytic bacteria (Kittiwongwattana and Thawai, 2015), flower induction (Khurana *et al*., 2011) and environment toxicity (Charles *et al*., 2006) in duckweeds. Despite these recent advances, currently, no stable transformation protocol and genetic manipulation tools have been established in *L. aequinoctialis*.

Here, we describe the optimization of a transformation protocol and the application of CRISPR/Cas9‐mediated targeted mutagenesis in *L. aequinoctialis*. We demonstrate that using *Agrobacterium* to transform the duckweed plants with a CRISPR/Cas9 vector can efficiently generate biallelic mutant plants in 5–6 weeks with high success rate.

## Results

### A stable and efficient genetic transformation system for *L. aequinoctialis*

**Figure 1:**
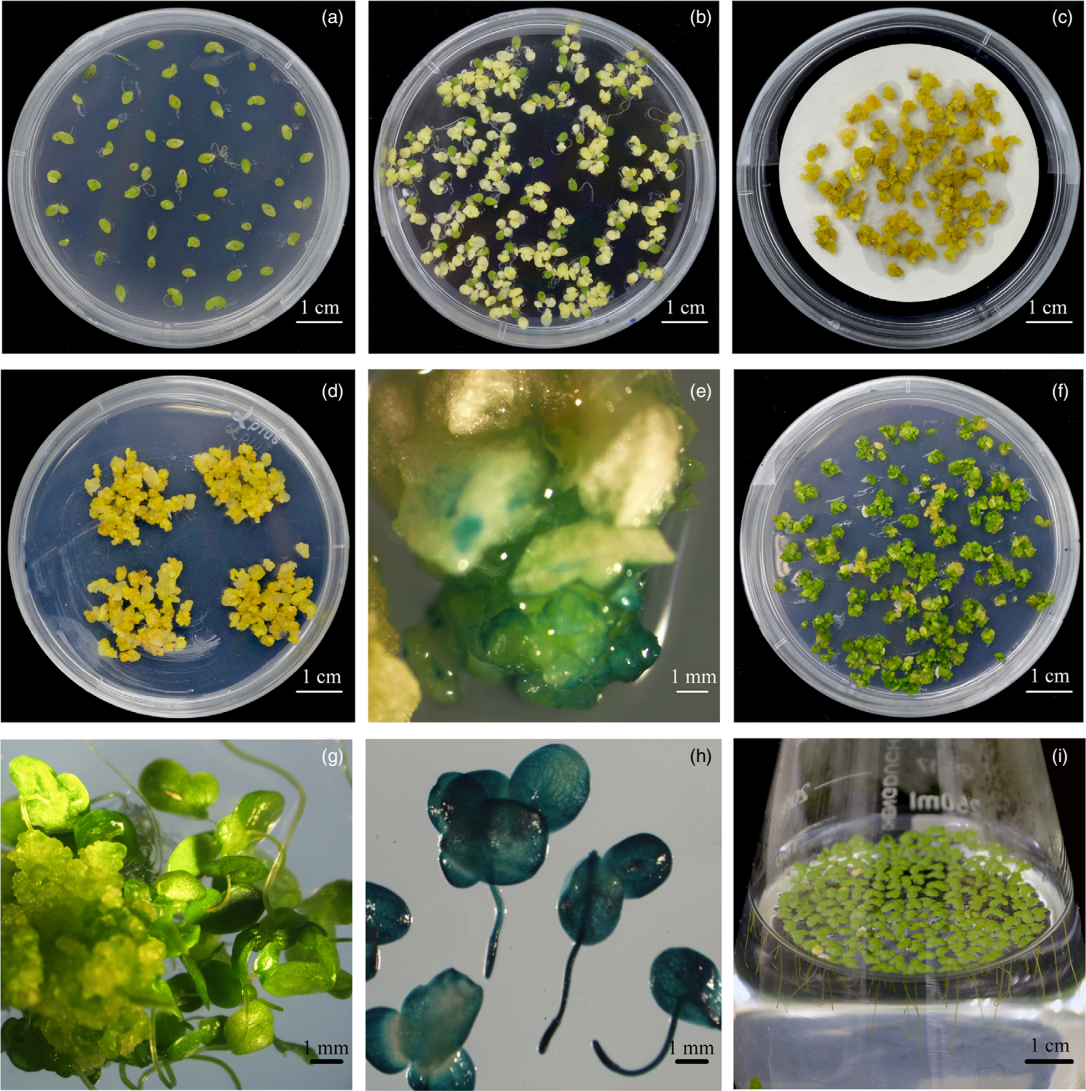
Stable efficient genetic transformation of *Lemna aequinoctialis*. (a) Frond sterile culture; (b) Callus induction; (c) *Agrobacterium tumefaciens* infection; (d) Callus screening; (e) GUS staining of callus; (f) Callus differentiation; (g) Frond regeneration; (h) GUS staining of transgenic frond after 70 days cultivation; and (i) Regeneration frond liquid cultivation.

To establish a stable and efficient genetic transformation system in *L. aequinoctialis* (Figure 1a–h), we optimized previous protocols in several steps. First, by using an available callus induction protocol (Cantó‐Pastor *et al*., 2015), we screened more than 100 clones of duckweed that were collected in China (Table S1). Among them, seven had more than 90% callus induction rate, and *L. aequinoctialis* 6002, which showed highest callus induction rate was used for further investigations. Second, we compared the infection efficiency among three different *Agrobacterium tumefaciens* strains that were used in previous studies: EHA105, AGL1 and GV1301. Among them, both the strain EHA105 and AGL1 showed overall high infection efficiency (Figure S2). We used EHA105 for establishing the transformation protocol in *L. aequinoctialis* as it is a strain widely used for plant transformation. To estimate the transformation rate, a GUS vector was used for the transformation. At 3 days after *Agrobacterium* inoculation, we found more than 94% (80 out of 85) of the calli expressed GUS (Figure 1e), suggesting the overall high infection rate. The transformed calli (survived in the selection medium) were then transferred to the regeneration medium (Figure 1f), and transgenic fronds were obtained after 4 weeks (Figure 1g). Measuring GUS signals in transgenic fronds at 70 days after the transformation suggests that all of the transformations are stable (Figure 1h). The whole transformation procedure (from callus infection to the establishment of transgenic fronds) took only 5–6 weeks.

The detected GUS signal could be due to T‐DNAs from living *A. tumefaciens*. To further examine whether the detected GUS signal was resulted from T‐DNAs from living *A. tumefaciens,* we first detected the *A. tumefaciens curdlan* gene via PCR. After 35 cycles of PCR, no gene signal found (Figure S4). Second, to examined whether the T‐DNAs were integrated into the duckweed genomic DNA, we performed thermal asymmetric interlaced PCR (TAIL‐PCR). Using the combinations of T‐DNA specific and degenerate primers, we examined four independently transformed lines (Figure S3). Sequencing four amplified DNA fragments confirmed that in three lines T‐DNAs were inserted into the region that showed high homology to *S. polyrhiza* genomic sequences (Table 1). In one line, the surrounding genomic sequence near the T‐DNA insertion showed no homology to any other sequences in the NCBI database, likely because the T‐DNA was inserted to a *L. aequinoctialis* species‐specific region (Table 1). These results showed that the observed GUS expression resulted from T‐DNAs that were integrated into the *L. aequinoctialis* genomes.

**Table 1:** *Agrobacterium tumefaciens*‐mediated transformation resulted in the integration of T‐DNA into the *L. aequinoctiali*s genome

Transformant	Border	Description	Total score	Query cover (%)	Sequence identity (%)	Accession no.
Tr1	RB	*Spirodela polyrhiza* strain 9509 chromosome 12 sequence	139	12	96	CP019104.1
Tr2	RB	No homology	–	–	–	–
Tr3	RB	*Spirodela polyrhiza* strain 9509 chromosome 3 sequence	176	22	91	CP019095.1
Tr4	RB	*Spirodela polyrhiza* strain 9509 chromosome 3 sequence	65.3	12	90	CP019095.1

Flanking sequences of transferred T‐DNA were obtained through thermal asymmetric interlaced polymerase chain reaction (TAIL‐PCR) and compared to the NCBI nucleotide sequence database. Tr1 and Tr2 are plants transformed with T‐DNA of the pANIC6B vector, and Tr3 and Tr4 are plants transformed with T‐DNA of the pYLCRISPR/Cas9‐MH vector. RB, right border of T‐DNA.

### CRSPR/Cas9 target selection and vector construction

**Figure 2:**
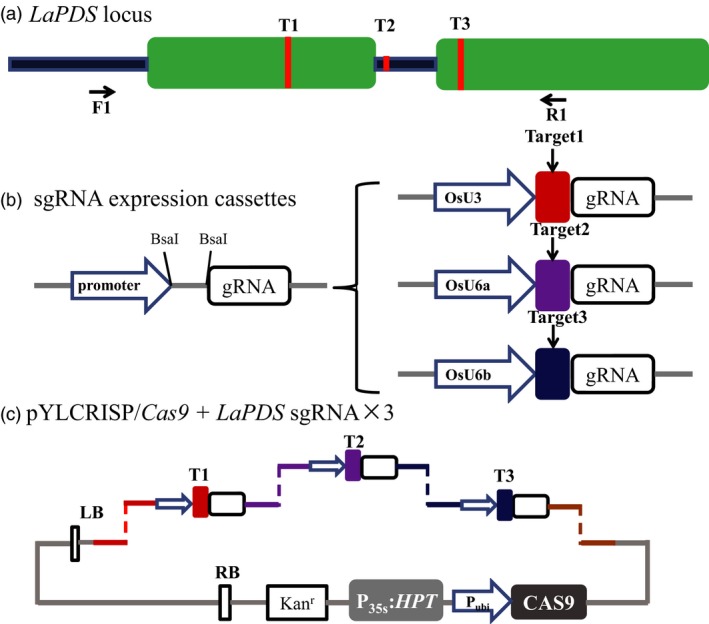
Schematic diagram of assembling Cas9/sgRNA construct and selecting target sites in the *LaPDS* gene. (a) Schematic illustrating the three small guide RNAs (sgRNAs, red lines) targeting the *LaPDS* coding sequence. Green boxes indicate exons, blue lines indicate introns. F1 and R1 indicate binding sites of the primers using for PCR amplification. (b) Schematic view of the method for constructing the expression cassettes of sgRNAs. Left, the backbone of sgRNA that any specific targeting sequence can be inserted between the promoter and the unchanged part of guide RNA using BsaI. Right, the three promoters from Rice, OsU3, OsU6a and OsU6b were used to drive the three *LaPDS* targeted sgRNAs, respectively. (c) Schematic diagram of the assembling of sgRNAs and Cas9 expression cassettes in a single binary vector for plant stable transformation mediated by *Agrobacterium*. By the design tails after cutting with BsaI, three sgRNA expression cassettes were ligated into the binary vector sequentially.

Using the optimized transformation system, we examined the efficiency of CRSPR/Cas9‐mediated genome editing in *L. aequinoctialis*. To this end, we focused on the duckweed phytoene desaturase gene (*LaPDS*), a common marker gene for testing genetic manipulation tools in plants (Fan *et al*., 2015; Shan *et al*., 2013). Based on the genomic sequence of *LaPDS* (Figure S1), we selected three targets (T1–T3, Table 2) using the CRISPR‐P 2.0 design tool and RNA Folding Form (Liu *et al*., 2017). While T1 and T3 are located in the first and second exon, respectively, T2 is located in the first intron (Figures 2a and S1). To increase the chance of obtaining a high sgRNA transcription, three promoter sequences from rice (a monocot species, same as duckweeds), OsU3, OsU6a and OsU6b, were used to drive the transcription of the sgRNA against T1, T2 and T3, respectively (Figure 2b). The expression cassettes including the three targets were inserted into the binary vector pYLCRISPR/Cas9‐MH using BsaI (Ma *et al*., 2015a). An overview of the constructed vectors is shown in Figure 2c. The CRISPR/Cas9 vector was transformed into the *L. aequinoctialis* calli using the optimized *Agrobacterium* transformation protocol.

**Table 2:** sgRNA sequences used in this study and their predicted sgRNA efficiency

Target site	Sequence	GC%	Predicted sgRNA efficiency
T1	TGATTCAAAGAAGCTGGAGCTGG	45	5.26
T2	CCACGAATCAGTTTTCTTCCGGA	45	7.01
T3	CCCGAGGCCACCATTAGAGGACA	55	7.29

The sgRNA efficiency was predicted via the CRISPR Efficiency Predictor (https://www.flyrnai.org/evaluateCrispr/). The sequence with underline is the sgRNA. The other three nucleotides without underline are the PAM sequence.

### Generating CRISPR/Cas9‐mediated mutagenesis in *L. aequinoctialis*

**Figure 3:**
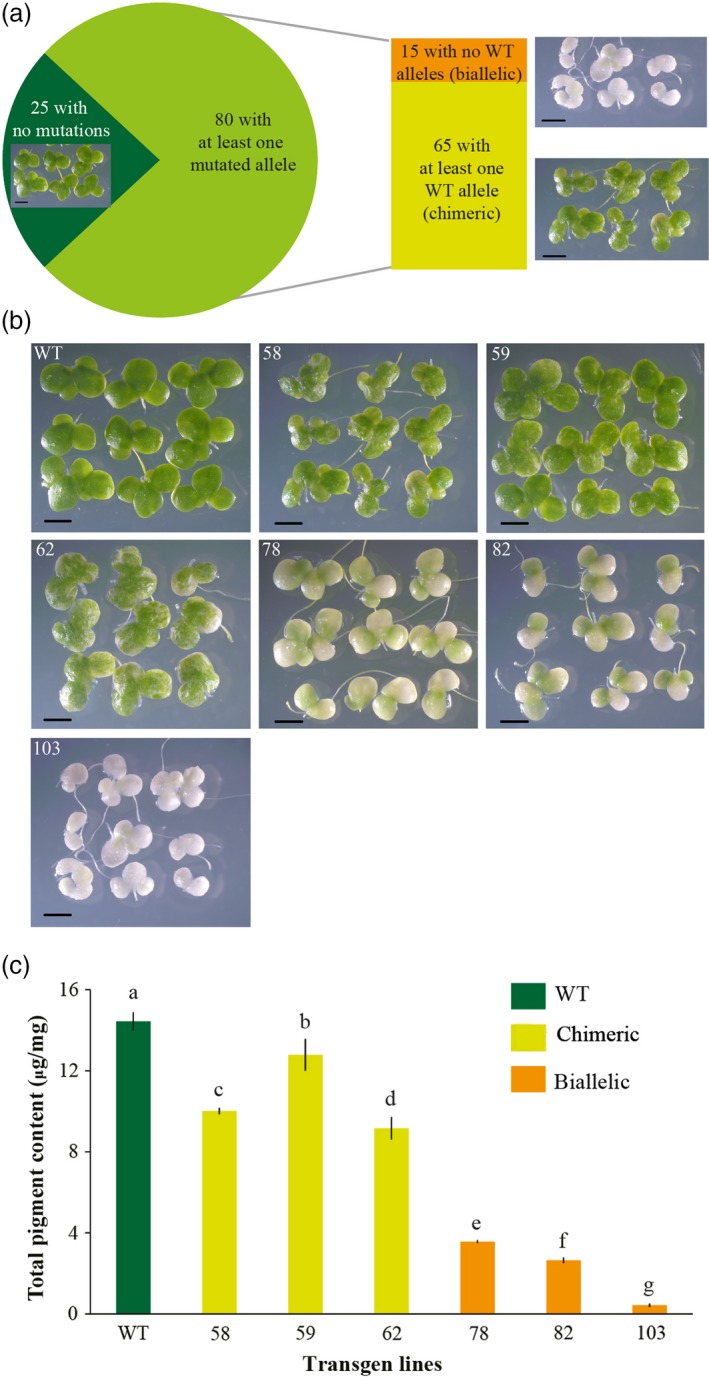
The efficiency of the CRISPR/Cas9 system in *Lemna aequinoctialis*. (a) among 105 transformed lines, 80 of them contained at least one mutated allele. Among these 80 mutated lines, 15 lines contained no WT alleles (all biallelic mutations) and 65 lines had at least one WT alleles (all chimeric mutations). Scale bars: 2 mm. The picture for each typic line was shown. (b) Albino phenotype of transgenic duckweed. 58, 59 and 62 are chimeric mutant lines; 78, 82 and 103 are biallelic mutant lines; Scale bars: 2 mm. (c) Total pigment content of different transgenic *Lemna aequinoctialis* lines. Data are means of three replicates and error bars indicate standard deviations. Different letters indicate significant difference among the transgenic lines (ANOVA, Duncan's test; *P* < 0.05).

To examine the efficiency of the constructed CRISPR/Cas9 vector in *L. aequinoctialis*, we amplified and sequenced the *LaPDS* gene from the 105 independently regenerated plants. In total, while 25 plants contained only WT alleles, 80 plants contained at least one mutated allele (Figure 3), indicating a mutation success of 76.2%. Among these 80 mutated plants, 15 showed no WT allele, suggesting that *LaPDS* was completely mutated (Figure 3). Interestingly, all of the 15 plants that contained no WT allele were biallelic mutants, in which two different mutated alleles were found. Similarly, all of the 65 plants that contained at least one WT alleles contained also at least two additional mutated alleles (chimeric mutants). Consistently, biallelic mutants showed an albino phenotype and significantly less chlorophyll *a*, chlorophyll *b* and total carotenoids, and the chimeric mutants showed slightly lower levels of pigments (Figure 3 and Table S3).

Some of the biallelic mutants still showed a slightly green colour (Figure 3), which can be due to a small amount of cells that still contain WT allele or other unknown mechanisms. To further check the presence/absence of WT alleles, we designed WT allele‐specific primers to amplify the genomic DNA of the two selected biallelic mutants. No PCR product can be found, which suggests that the slightly green colour of the biallelic mutants is likely not due to the presence of wide‐type allele containing cells (Figure S6).

### Spectrum of CRISPR/Cas9‐mediated mutations in *L. aequinoctialis*

Analysing the mutation spectrum of the 105 transgenic lines showed that 76.2% of them had at least one mutation at the site T3, while 25.7% of them had at least one mutation at the site T2. No plants showed any mutation at the site T1 (Table 3). At the site T3, while 66 plants contained chimeric mutations, 14 had biallelic mutations (and showed albino phenotype). At the site T2, 26 plants contained chimeric mutations and one contained biallelic mutations (and showed an albino phenotype).

**Table 3:** Summary of mutations at each target site among all transformed plants

Target site	No. of lines analysed	No. of lines with mutation	Mutation frequency (%)	Genotypes		No. of lines with mutations in any target
				No. biallelic lines	No. chimeric (including WT allele) lines	
1	105	0	0	0	0	
2	105	27	25.7	1	26	
3	105	80	76.2	14	66	
						80

**Figure 4:**
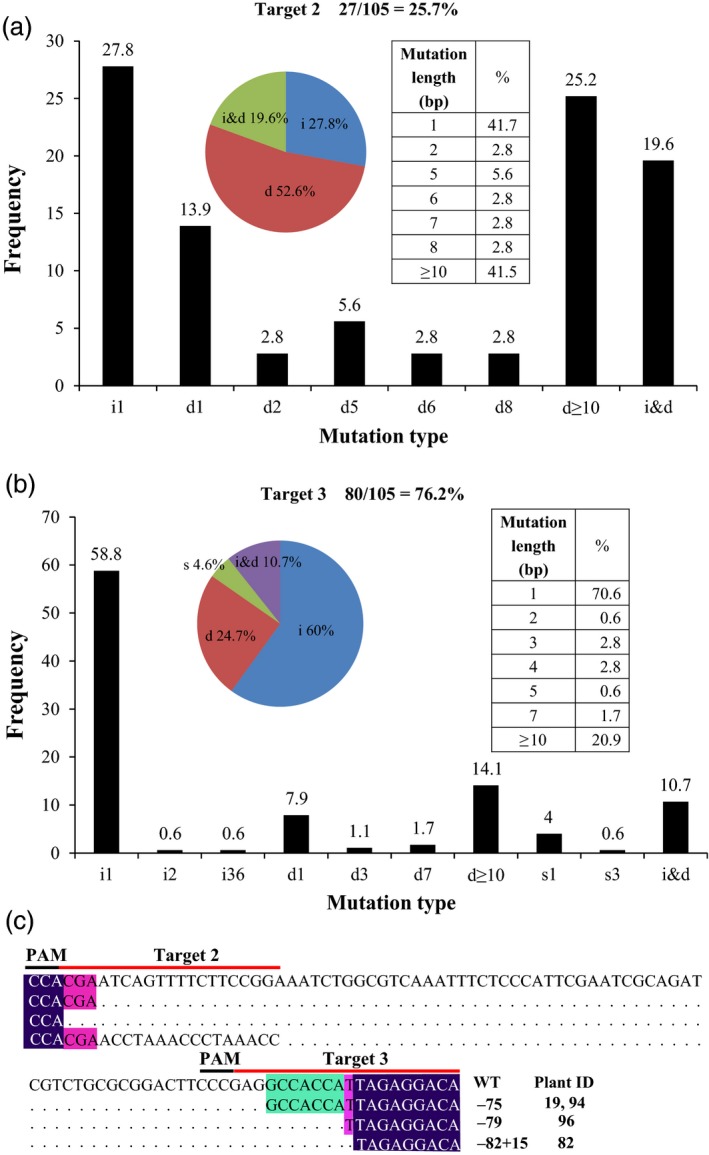
CRISPR/Cas9‐induced mutation types and frequency. (a) Mutation types and frequency in Target 2. (b) Mutation types and frequency in Target 3. (c) Large fragment deletions and insertions caused by combined mutagenesis at two target sites. Left insert in a and b refers to proportion of different mutation types: insertion (i), deletion (d), substitution (s) and the combination of insertion and deletion (i and d). Right insert in A and B refers to the mutation length frequency. In panel A and B, *X*‐axis refers to mutation type and length, *Y*‐axis refers to frequency.

Most of the mutations at the sites T2 and T3 are short insertions and deletions (Figures 4 and S5), with 1‐bp insertion as the most frequent mutation type. In addition, large insertions and deletions (more than 10 bps) were found. Interestingly, base substitutions (C→G, C→A, G→T, G→C, G→A and A→G) followed by insertions and deletions were also found (Figures 4 and S5). Furthermore, we observed that the plants of line #19, #82, #94 and #96 contained large deletions (>50 bps) due to combinational effects of the two target sites (Figures 4 and S5). Together, these results demonstrate that CRISPR/Cas9 can be used to efficiently create different types of mutations in *L. aequinoctialis*.

## Discussion

Here, we report an efficient genetic transformation system and for the first time demonstrated that CRISPR/Cas9 can be an effective tool for genome editing a duckweed species. These tools will significantly accelerate duckweed research, both for understanding basic plant biology and for industrial applications.

An efficient genetic transformation protocol is essential for high‐throughput genetic manipulations in plants. Although stable genetic transformation methods have been used in different duckweed species, the efficiency varied among species (Table S2). Here, by optimizing the transformation protocol, we achieved a 94% transformation success rate, which is higher than previous reports on duckweed transformations. For example, in *L. minor*, a different species of the same genus, the efficiency obtained was approximately 10% (Chhabra *et al*., 2011), 40% (Yang *et al*., 2018a) and 59% (Cantó‐Pastor *et al*., 2015). Furthermore, the whole transformation only took 5–6 weeks, which is similar to the fastest protocol that was previously reported in *L. minor* (Cantó‐Pastor *et al*., 2015), and the genetic transformation was stable in the regenerated plants for at least 70 days (~30 generations).

In comparison to previous *Agrobacterium*‐mediated transformation protocols in *L. minor* (Cantó‐Pastor *et al*., 2015; Chhabra *et al*., 2011; Firsov *et al*., 2015, 2018; Ko *et al*., 2011; Sun *et al*., 2007; Yamamoto *et al*., 2001; Yang *et al*., 2013, 2017, 2018a), the increased efficiency of our protocol is likely resulted from adopting sonication coupled with vacuum infiltration infection method. In several previous studies, such as banana (Subramanyam *et al*., 2011), chickpea (Indurker *et al*., 2010) and cowpea (Bakshi *et al*., 2011), it has been shown that the combination of vacuum infiltration and sonication can greatly improve the efficiency of *Agrobacterium* infection. Although it remains to be tested, we believe that this infection method can also be used to improve the transformation efficiency in other duckweed species.

A major limitation of our transformation protocol is that the success rate might be highly dependent on the choice of the genotypes, which can vary in their callus induction rate (Table S1). A similar effect was also found in *L. minor* (Yang *et al*., 2018a), indicates that this might be a general pattern in species of the genus *Lemna*. To resolve this issue, two possible approaches can be applied. First, in addition to the conventional callus‐based transformation, previous studies showed that a direct transformation of fronds (Ko *et al*., 2011; Yang *et al*., 2018a) or clusters (the specific structure for *Wolffia*) (Heenatigala *et al*., 2018; Khvatkov *et al*., 2015, 2018) can work in duckweeds. Therefore, for the genotypes in which callus induction rate is low, direct frond transformation, which worked for different tested genotypes of *L*. *minor* (Yang *et al*., 2018a), can be used. The disadvantage of direct frond transformation is that the whole process requires ~12 weeks, which is more than two times longer than the callus‐based transformation protocol. Second, an optimized callus induction protocol can be used. Recently, a study showed that optimizing the callus induction medium can significantly increase the rate of callus induction in *S. polyrhiza* (Wang, 2016). Our preliminary results also showed that using the optimized medium reported from Wang (2016) can result in 100% callus induction among all 16 tested *S. polyrhiza* genotypes. It seems plausible that the optimization of the callus induction medium can be used to increase the callus induction efficiency also in other *Lemna* species.

The efficient and stable genetic transformation system in *L. aequinoctialis* enabled us to establish the CRISPR/Cas9‐based genome editing tool in this species. As a proof‐of‐concept, we targeted three sites of the *LaPDS* gene in *L. aequinoctialis*. Among the 105 regenerated plants from the transformed calli, 80 of them contained mutated alleles, of which 15 were biallelic mutants (have no WT alleles), 65 were chimeric mutants (at least one WT alleles and two additional mutated alleles). This represents an average of 14.3% biallelic mutant generation rate. Because under normal conditions, *L. aequinoctialis* plants reproduce through asexual budding, the biallelic mutants will be completely maintained in the plant genome over the generations, thus the plants and their offspring can be directly used for analysing gene functions or further bioengineering.

The relatively high frequency of chimeric mutants could arise due to two non‐exclusive reasons. First, it could be due to different independent CRISPR/Cas9‐mediated mutagenesis among different cells. Since CRISPR/Cas9 is integrated into the plant genome, the mutagenesis machinery should be continuously active at different stages of plant regeneration and development. When the mutagenesis took place in the first embryogenic cell, all cells in the regenerated plant should contain the same allele at the targeted locus, resulting in heterozygous or biallelic mutants. However, when the mutagenesis took places in cells after the first embryogenic cell division, the regenerated plants may contain cells with independently mutated alleles (chimeric). Second, the observed chimeric mutants may also arise if some of the regenerated *L. aequinoctialis* plants are polyploid. In this scenario, even the CRISPR/Cas9‐mediated mutagenesis took place in the first embryogenic cell, it may still result in more than two alleles at the targeted locus. Future studies that directly measure the ploidy level (e.g. using flow cytometry) of the regenerated plants are required to distinguish these two possible reasons.

*Phytoene desaturase (PDS)* encodes one of the key enzymes in the carotenoid biosynthesis pathway (Qin *et al*., 2007). We found that *L. aequinoctialis* plants in which *PDS* were completely disrupted showed albino phenotype (Figures 3) which is consistent with a previous report in *Arabidopsis* (Qin *et al*., 2007). Loss‐of‐function of *PDS* in *L. aequinoctialis* likely have disrupted the biosynthesis of carotenoid, which indirectly destroyed chlorophyll, as well as many other genes involved in carotenoid, chlorophyll and GA biosynthesis pathways (Qin *et al*., 2007). As a consequence, these biallelic mutants showed albino phenotype.

Analysing the CRIPSR/Cas9‐mediated mutagenesis showed that the gene editing efficiency varied substantially among target sites. For example, no mutations were found at the site T1. Because different promoters were used to drive different sgRNAs (Ma *et al*., 2015a), the capacity in driving the expression of sgRNA might have resulted in the observed differences. In addition, the sgRNA efficiency of T1 site was lower in comparison to the other two sites (Table 2), which might also have contributed to the low mutation efficiency at this site. These factors should be considered for future applications of the CRIPSR/Cas9 system in duckweeds.

A major concern of CRIPSR/Cas9‐mediated genome editing is the off‐target effects. Although several online tools have been developed to reduce the off‐targets effects (Bae *et al*., 2014; Liu *et al*., 2017), it requires complete genomic information, which is only available for *S. polyrhiza* (Hoang *et al*., 2018; Michael *et al*., 2017; Wang *et al*., 2014). Except sequencing the complete genome of the mutant plants, which is still relatively expensive due to the large genome size (>600 Mb), it remains unclear how many off‐targets were generated by the CRISPR/Cas9 in our mutant plants. To further evaluate the off‐target effects and optimize the CRISPR/Cas9 system in duckweeds, *S. polyrhiza*, which has a relatively small genome size (158 Mb), high‐quality reference genome and low mutation rates (Michael *et al*., 2017; Xu *et al*., 2019), would be an ideal system.

Together, we present the first CRISPR/Cas9 study in a duckweed species, which is one of the important future crops. We demonstrated that CRISPR/Cas9 coupled with an optimized transformation protocol can efficiently generate biallelic mutant lines in duckweed within 5–6 weeks, which is significantly shorter than the currently available protocols in model plants such as *Arabidopsis* and rice (Ma *et al*., 2015a; Shan *et al*., 2014; Zhang *et al*., 2014). Together with the rapidly accumulating genomic information in duckweeds, we anticipate that the established gene manipulation tools will boost duckweed researches and pave the way to fully utilize the remarkable duckweed plants for both industrial applications and researches on the mechanisms of plant–environment interactions.

## Materials and methods

### Plant material and callus induction

A duckweed stock collection stock was established containing more than 100 accessions. The duckweed species was identified by *atpF‐atpH* noncoding spacer (Wang *et al*., 2010) and registered in the Duckweed Stock Cooperative (http://www.ruduckweed.org/database.html). Plants were cultivated for 2–3 weeks in Schenk and Hildebrandt medium (SH) with 1% (w/v) sucrose at pH 5.6 (Schenk and Hildebrandt, 1972). Fronds were maintained at 25 °C under a 16 h photoperiod of approximately 100 μmol/m^2^/s of white light. Fronds were incubated on induction medium containing Murashige and Skoog basal salts, 3% (w/v) sucrose, 4.52 μm 2,4‐dichlorophenoxyacetic acid (2,4‐D), 0.45 μm thidiazuron (TDZ) and 7.9 g/L bacteriological agar at pH 5.9. The cultures were kept in the dark at 24 °C. Embryogenic calli were obtained after 3–4 weeks of culture. The developed calli were propagated on the fresh induction medium for another 4–5 weeks before they were used in transformation assays.

### *Agrobacterium* preparation and genetic transformation

We compared the infection efficiency of *Agrobacterium tumefaciens* using EHA105, AGL1 and GV1301. Single colonies of *A. tumefaciens* were transferred to liquid LB medium containing 50 mg/L kanamycin and 25 mg/L rifampicin. The cultures were grown at 28 °C with shaking (200 rpm) until the OD_600_ reached 0.8–1.0. Freshly prepared 100 μm acetosyringone (3′,5′‐dimethoxy‐4′‐hydroxyacto phenone, ACROS Organics, Morris Plains, NJ, USA) was added to the cultures, and the mixture was continuously shaken for another 2 h. Cells were then pelleted by centrifugation at 2400×***g*** for 15 min and resuspended in the induction medium (same as mentioned above). The density (OD_600_) of the resuspended *Agrobacterium* was adjusted to approximately 0.4. The GUS expression vector pANIC6B (Mann *et al*., 2012) was introduced into the *Agrobacterium* strain EHA105 and used for genetic transformation.

Embryogenic calli of *L. aequinoctialis* were immersed in the *Agrobacterium* suspension in 350‐mL tissue culture bottles. The bottles were placed in a vacuum chamber and vacuum was applied for 10 min. Then, the bottles were placed in a bath‐type sonicator (Branson ultrasonic cleaner CPX2800, Branson Ultrasonics Corp., Danbury, CT, USA) and then subjected to ultrasound at a frequency of 40 kHz for 5 min at 17 °C. After sonication, the bottles were vacuum infiltrated again for 10 min. After releasing the vacuum, the callus pieces and *Agrobacteria* were incubated for 30 min with gentle shaking. Excess bacteria were removed after the incubation by transferring the infected callus pieces onto filter papers wetted with induction medium and placed in empty petri dishes in the dark at 25 °C for co‐cultivation.

### Selection and regeneration of transgenic plants

Three days after co‐cultivation, the infected calli were transferred onto regeneration medium: Gamborg's B5 basal medium supplemented with 4.65 μm kinetin, 2.57 μm indole‐3‐acetic acid (IAA), 1% (w/v) sucrose, 9.48 μm hygromycin (PhytoTechnology Laboratories, Shawnee Mission, KS, USA), 600 μm timentin and solidified with 7.9 g/L agar. After 1 week on the agar in dark, the calli were cultivated under a 16 h photoperiod of approximately 50 μmol/m^2^/s of white light for 4 weeks. The regenerated fronds were transferred onto a conservation medium: SH medium supplemented with 0.6% (w/v) sucrose, 300 mm timentin and solidified with 7.9 g/L agar. Regenerated fronds were proliferated on liquid SH medium.

### GUS staining of nodules and fronds

GUS activity in co‐cultivated nodules and regenerated fronds was monitored throughout the transformation experiments. GUS assay was performed by incubating the nodular calli and fronds in histochemical buffer (0.1 m sodium phosphate buffer, pH‐7.0, 50 mm EDTA, 0.5 mm K3 Fe(CN)6), 0.5 mm K4 Fe(CN)6, 0.1% Triton‐X‐100 and 1 mg/mL X‐gluc (5‐bromo‐4‐chloro‐3‐indolyl‐β‐glucuronidase) overnight at 37 °C (Jefferson, 1987). After incubation, nodular calli and fronds were washed with deionized water and cleared with 95% ethanol prior to observations using a stereomicroscope (Nikon SMZ‐U, Nikon, Inc., Melville, NY, USA).

### Cloning of the *LaPDS* gene

The DNA fragment of *LaPDS* was amplified with gene‐specific primers (Table S4), which were designed based on the gene sequence obtained from the *L. aequinoctialis* transcriptome sequences (Yu *et al*., 2017). PCR reactions were carried out with *Pfu* DNA polymerase (Takara, China) in a total volume of 50 μL contained 1× buffer, 0.2 mm (each) dNTPs, 0.4 μm primers, 5 U *Pfu* DNA polymerase and 50 ng of template DNA. PCR cycling parameters were: denaturation at 95 °C for 3 min; 35 cycles of 95 °C for 45 s, 56 °C for 30 s and 72 °C for 120 s, followed by a final extension of 72 °C for 10 min. PCR products were cloned into pMD18‐T Vector (Takara, China) and sequenced.

### CRISPR/Cas9 target site selection

Target sequences were designed within the *LaPDS* gene using the online tools CRISPR‐P 2.0 (http://cbi.hzau.edu.cn/crispr/) (Liu *et al*., 2017). To further selected target sequence, secondary structure analysis of target‐sgRNA sequences was carried out with the program RNA Folding form (http://mfold.rna.albany.edu/?q=mfold/ RNA‐Folding‐Form2.3) (Zuker, 2003). Target sequences were further selected to avoid those paired to the sgRNA with more than six continuous bp. On the other hand, target site near the 5′ end of *LaPDS* gene was the preferred target to increase the gene targeting effect. The efficiency score of sgRNA was predicted by CRISPR efficiency predictor (Housden *et al*., 2015).

### Assemble Cas9/sgRNA construct and plant transformation

A CRIPSR/Cas9 construct carrying three sgRNA cassettes was generated using the binary pYLCRIPSR/Cas9 multiplex genome targeting vector system provided by Yao‐Guang Liu of South China Agricultural University (Ma *et al*., 2015a). Three plasmids with sgRNA cassettes driven by OsU3, OsU6a and OsU6b, respectively, were assembled according to the golden gate cloning protocol.

The CRISPR/Cas9 constructs were introduced into *A. tumefaciens* strain EHA105 by electroporation as previous described (Ma *et al*., 2015a). Transformation of duckweed and stable genetic transformation validation was performed as described above.

### Validation of stable transformations

We validated stable genetic transformations by analysing the integration of T‐DNA into the *L. aequinoctialis* genome. To identify the insertion sites of the T‐DNA, thermal asymmetric interlaced PCR (TAIL‐PCR) was performed as previously described (Liu and Chen, 2007; Wang *et al*., 2011). To further examine the presence/absence of living *Agrobacteria* in the regenerated tissue, the *A. tumefaciens* strain EHA105 *curdlan* gene was amplified by PCR with the gene‐specific primers for 35 cycles, *EHA105‐crds‐*F and *EHA105‐crds‐*R (Table S4). The PCR conditions were same as described above.

### Genomic DNA extraction and detection of CRISPR/Cas9‐mediated mutations

The genomic DNA was extracted from both transgenic and wild‐type plants following the CTAB method (Porebski *et al*., 1997). For each plant, ~100 mg fresh frond was ground in liquid nitrogen and 400 μL of pre‐heated CTAB buffer was added. After incubating at 65 °C for 30 min, 200 μL of chloroform was added and the resulting mixture was kept at room temperature for 10 min. After centrifugation at 16 000 ***g*** for 5 min, the supernatant was transferred to a new tube, mixed with 300 μL of isopropanol and incubated at 4 °C for 30 min. Then, genomic DNA was precipitated by centrifuge at 16 000 ***g*** for 10 min, the supernatant was removed and the DNA pellet was washed with 0.5 mL of 70% ethanol. The genomic DNA pellet was dissolved in 100 μL of H_2_O, and the concentration was determined using a spectrophotometer (NanoDrop 2000, Thermo Fisher Scientific, Inc., Pittsburgh, PA, USA).

The extracted genomic DNA was then used as a template to amplify the endogenous *PDS* fragment by PCR. PCR was performed using the specific primers, *LaPDS*‐F1 and *LaPDS*‐R1 (Table S4), which covered the region of target site 1, 2 and 3. The PCR products were sequenced directly using the specific primers with Sanger‐sequencing approach. Biallelic and chimeric mutations that produced superimposed sequence chromatograms from direct sequencing were decoded using the Degenerate Sequence Decoding method (Ma *et al*., 2015b). Biallelic mutants showed two distinct allelic mutations, but no wild‐type allele, while chimeric mutations had more than two distinct allelic mutations and an additional wild‐type allele. PCR products from all biallelic mutants and some chimeric mutants were cloned into the pMD18‐T Simple vector (Takara, China), and ten to twenty clones for each sample were sequenced using the same method mentioned above. DNAMAN (version 9; Lynnon Biosoft, Inc., San Ramon, CA) was used for sequence alignment analysis. Based on the deletion information obtained from the two biallelic mutant #34 and #78, wt *LaPDS* allele‐specific primers were designed(34‐F and 34‐R; 78‐F, 78‐R) (Table S4) to examine the presence of wt cells in the transgene lines.

### Chlorophyll and total carotenoids quantification

Chlorophyll *a*, chlorophyll *b* and total carotenoids were extracted in ethanol and quantified with spectrophotometry (GeneQuant 1300, GE Healthcare, San Diego, CA, USA) using three biological replicates (Lichtenthaler and Wellburn, 1983).

### Statistical analysis

Data were presented as the mean ± standard deviation of the mean of triplicate samples. Significant differences between means of WT and transgenic lines were determined using one‐way analysis of variance followed by Duncan's multiple‐range tests, using the SPSS statistical package (version 16.0; SPSS Inc., Chicago, IL, USA) at a significance level of *P *< 0.05.

## Conflict of interest

The authors declare no conflicts of interest.

## Author contributions

Y.B. Ma and G.K. Zhou designed the study. Y. Liu and Y. Wang contributed to the experiments. Y.B. Ma, Y. Liu, Y. Wang and X.F Tang performed data analysis. C.J. Yu, G. He, H. Xu, Y.L Tang, S.M Wang, C.X Fu, J.S Zhao and S. Xu assisted with the data analysis. Y.B. Ma, S. Xu and G.K. Zhou wrote the manuscript. All of the authors approved the final manuscript.

## Supporting information


**Figure S1** Genomic sequence of the *LaPDS*.
**Figure S2** Comparison of transformation efficiency among three different *Agrobacterium* strains based on GUS staining.
**Figure S3** Analysis of T‐DNA insertions.
**Figure S4** Examine living presence/absence of *Agrobacterium* in the regenerated tissue.
**Figure S5** Different types of mutations detected in the transgenic duckweed after CRISPR/Cas9‐mediated gene editing. The plant numbers are shown in black.
**Figure S6** Examine presence/absence of WT allele containing cells in two biallelic transformants.
**Table S1** Screening *L. aequinoctialis* strains with high callus induction rate.
**Table S2** Stable genetic transformation methods among different duckweed species.
**Table S3** Chlorophyll *a*, chlorophyll *b* and total carotenoids content in transgenic plants.
**Table S4** Primers used in this study.
