# Supplementary material for: Efficient genetic transformation and CRISPR/Cas9‐mediated genome editing in Lemna aequinoctialis
Source: Plant Biotechnol J. 2019 May 3;17(11):2143–52. doi: 10.1111/pbi.13128 (PMC6790374; doi:10.1111/pbi.13128)
Supplement: Supplementary file 1 — Figure S1 Genomic sequence of the LaPDS. Figure S2 Comparison of transformation efficiency among three different Agrobacterium strains based on GUS staining. Figure S3 Analysis of T‐DNA insertions. Figure S4 Examine living presence/absence of Agrobacterium in the regenerated tissue. Figure S5 Different types of mutations detected in the transgenic duckweed after CRISPR/Cas9‐mediated gene editing. The plant numbers are shown in black. Figure S6 Examine presence/absence of WT allele containing cells in two biallelic transformants. Table S1 Screening L. aequinoctialis strains with high callus induction rate. Table S2 Stable genetic transformation methods among different duckweed species. Table S3 Chlorophyll a, chlorophyll b and total carotenoids content in transgenic plants. Table S4 Primers used in this study. [file PBI-17-2143-s001.docx]

**Supporting information**

**Supplementary Figure S1.** Genomic sequence of the *LaPDS*. The capital is the exon, the blue lowercase is the intron, and the red lowercase is the promoter.

cgtccctctctctctctctctctctctctctctctctgagtagcgtcttccactgggtggggagctgcgaattgggaccgcggggctattccgagctctctctgcgcgttcactcacagcacaagtacgcctgttccttccccttgttgccctacgatatcggttcatctatcatcgacagtcggagcactcgcccctcgcccactttcgttcctgcccgccgccggctccttcacccttttcagtttccctggcttttcagggaaacccgcaggtgaacATGGGATACACCGGCTCCGTCTCCGCCGTGAACACGGGCGGCGTGATTCAAAGAAGCTGGAGCTGGAATTCCAGCATCTCCAGGAAACTCCAATTCTCTTTCCGTAGCAGTAATCTTCTCGGCGACCGCCTAACTGTCGGGAGCGCATCGATTGCGGCCAAAACCCCTTCCTCGGGACGCTTGCAGgttcgagatccacgaatcagttttcttccggaaatctggcgtcaaatttctcccattcgaatcgcagATCGTCTGCGCGGACTTCCCGAGGCCACCATTAGAGGACACGATCAATTTCGTCGAAGCCGCCGCCCTATCCGCCTCTTTCCGAAGCTCCCCCCGTCCCTCCAAACCCCTCGAAGTCGTCATTGCGGGAGCAGGTCTGGCGGGTCTCTCCCTGGCAAAATATCTCGCCGACGCCGGCCATAAACCCATTTTGCTCGAAGCAAGGGAAGTCCTGGGAGGAAAGgtcgacccaccccgacaataacaacaaaatttaccctcttccatcttcgatctgacccatcctcttcctttcagCTCGCCGCATGGAAGGACAGCGACGGAGACTGGTACGAAACGGGTCTCCATATCTTCTgtaagtaatcgatgcaagaatgatctcgattcatcaagagattgaatggttccgtttgcatatagTCGGAGCATATCCGAACATGCATAATCTATTCGGGGAACTGGGGATCAACGACCGACTGCAATGGAAGGAGCACTCCATGATCTTCGCCATGCCCGACAAGCCCGGAGAATTCAGCCGCTTCGATTTTCCAGAGATTCTTCCATCCCCCATAAACGGtgatgtgctctccgtcttacattcctgtaatttttctattaatagttgaatttcaggAATCTGGGCGATTCTGAGGAACAACGAAATGCTGACGTGGCCCGAGAAGGTGCGATTCGCTATTGGGCTTCTCCCGGCCATGTTGGGCGGCCAGCCCTACGTGGAGGCCCAGGATGGCATAACCGTCAAAGATTGGATGAAGCGGCAGGGCGTGCCCGACAGAGTCAACGACGAGGTCTTCATAGCGATGTCAAAAGCTCTCAACTTTATCAATCCAGACGAGCTTTCCATGCAGTGCATCCTCATCGCACTTAACCGTTTTCTCCAGGttggactcctttaaattggaataataatgattagtatgttttgcttaaatttgttaatttttgtgtaaaaaaggAGAAACACGGGTCAAAGATGGCGTTCTTGGATGGGAATCCGCCAGAGAGGTTGTGCATGCCGATCGTTGACCATATTCAGTCACTGGGGGGTCGAGTGGAAGCAAACTCTCGGATACTGAAGATTGAGCTGAATGATGATAACACGGTGAAGCAGTTTGTTCTCGCTAATGGGAACGAAATTAGAGGGGATGCTTTTGTTTTCGCAACTCCAGTTGACATATTGAAGCTGCAGTTGCCTCCGAGCTGGAAGGAAATTCCCTACTTCAAGCGGCTGGATAAGTTGGTGGGAGTTCCGGTGATTAATGTCCACATCTGGTTTGACAGAAAATTGAAGAATACCTACGATCATCTGCTTTTTAGCCGGAGCCCTCTCTTGAGCGTGTATGCTGATATGTCAGTTACCTGCAAGGAGTACTACGACCCAAACAGATCAATGCTGGAGCTAGTTTTTGCACCGGCGGAGGAGTGGATCGGGAGGAGCGATAGCGACATCATTGACGCCACCATGGAAGAATTGGCCCGGCTCTTCCCTGACGAGATCGCCGCCGATCAGAGCAAGGCCAAGATACTCAAATCCCGCGTCGTCAAGACCCCGAGGTaaccaaatcttattttcgtttgtaaaaactatagtttagcgtatcaactgatatagaaatgttggtcttctcaaaggtCCGTGTACAAGACGGTGCCCGGGTGCGAACCCTGTCGCCCCCTTCAGAGGTCTCCAATCGAAGGCTTCTACTTGGCTGGCGACTACACCAAGCAAAAGTACTTGGCTTCCATGGAAGGTGCTGTTCTCTCTGGCAAGCTCTGCGCTCAGGCCATCCTCCAGGACTACGAGTTGTTGCAAGCTCGGAGAAGCAGGATGAGCTCTAAGGTCGAACCAGCCGTGGCCTAG


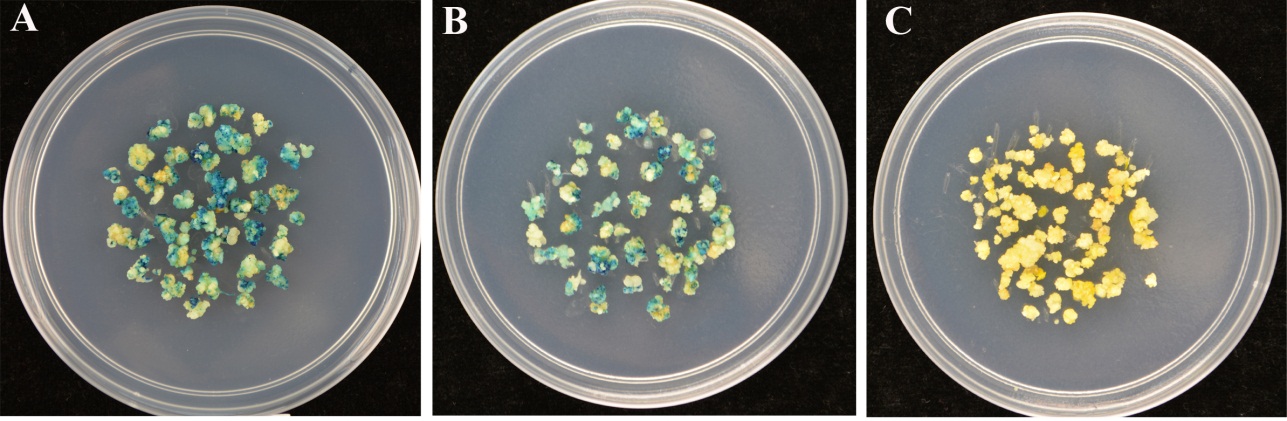


**Supplementary Figure S2.** Comparison of transformation efficiency among three different *Agrobacterium* strains based on GUS staining. A, EHA105; B, AGL1; C, GV1301.

**
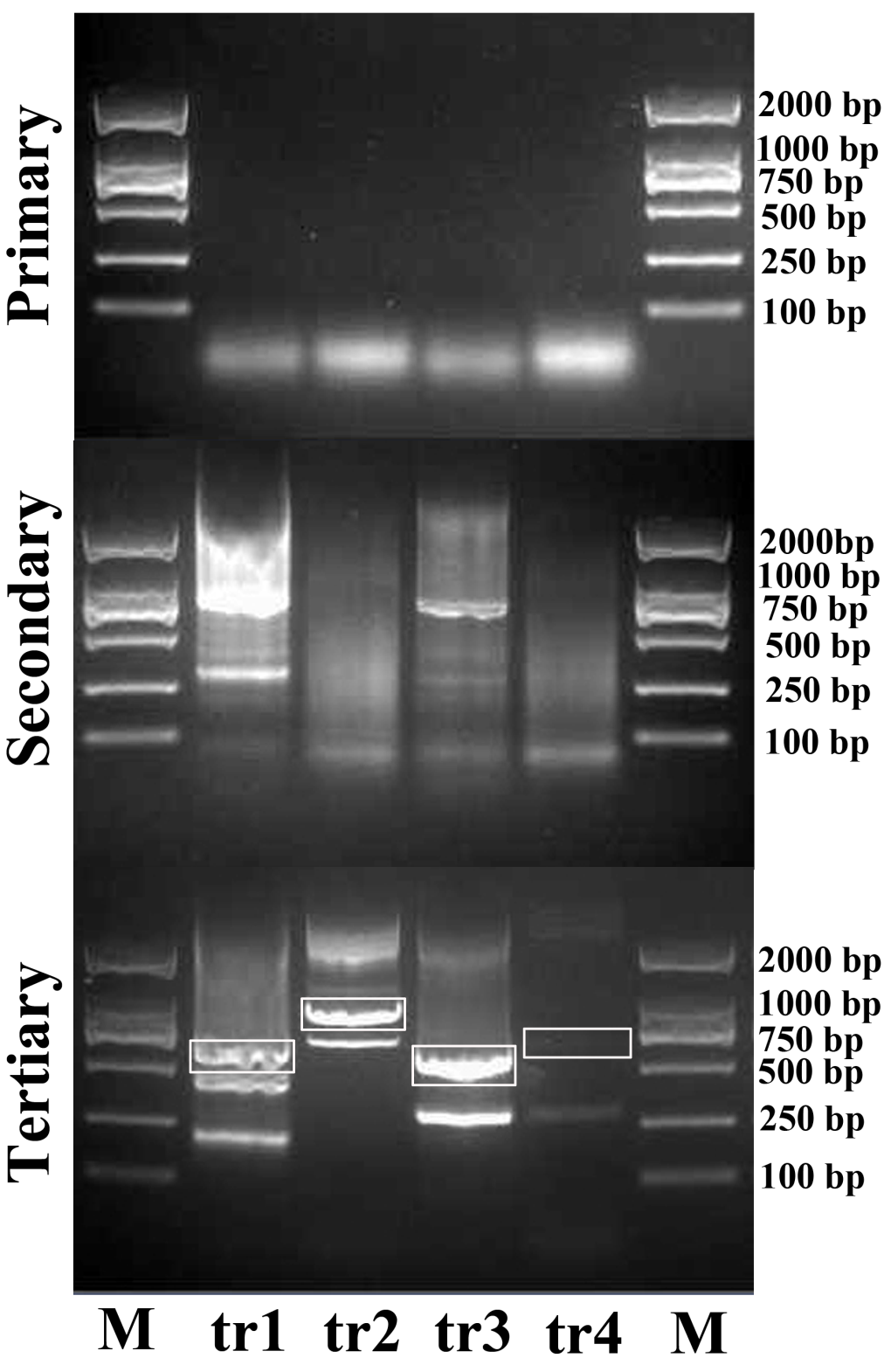
**

**Supplementary Figure S3.** Analysis of T-DNA insertions. The transformants were infiltrated with the T-DNA of plasmids pANIC6B (tr1 and tr2) and pYLCRISPR/Cas9-MH (tr3 and tr4). Amplifications were carried out with T-DNA specific primers and degenerate primers. Gels show the PCR products obtained after the first, second and third PCR reactions. The bands highlighted with boxes were extracted cut from the gel and sequenced. M: molecular weight marker DL2000 (Takara); tr1-tr4: PCR products from genomic DNAs extracted from different transformants, tr3 (#82) and tr4 (#103) are biallelic mutants.

**
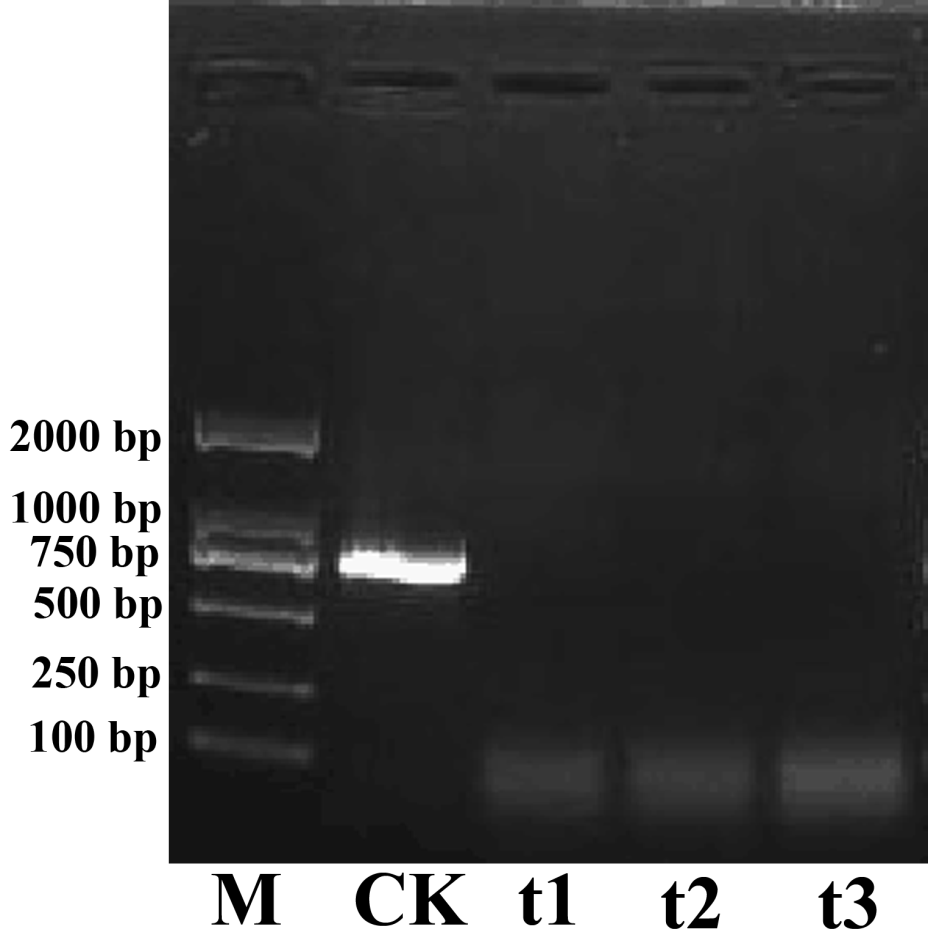
**

**Supplementary Figure S4.** Examine living presence/absence of *Agrobacterium* in the regenerated tissue. Amplifications were carried out for 35 cycles with specific primers of the *Agrobacterium* *curdlan* gene (*EHA105-crds-*F and *EHA105-crds-*R). Lanes: M, molecular weight marker DL2000 (Takara); CK, *Agrobacterium* DNA control; t1-t3, genomic DNA of different transformants.


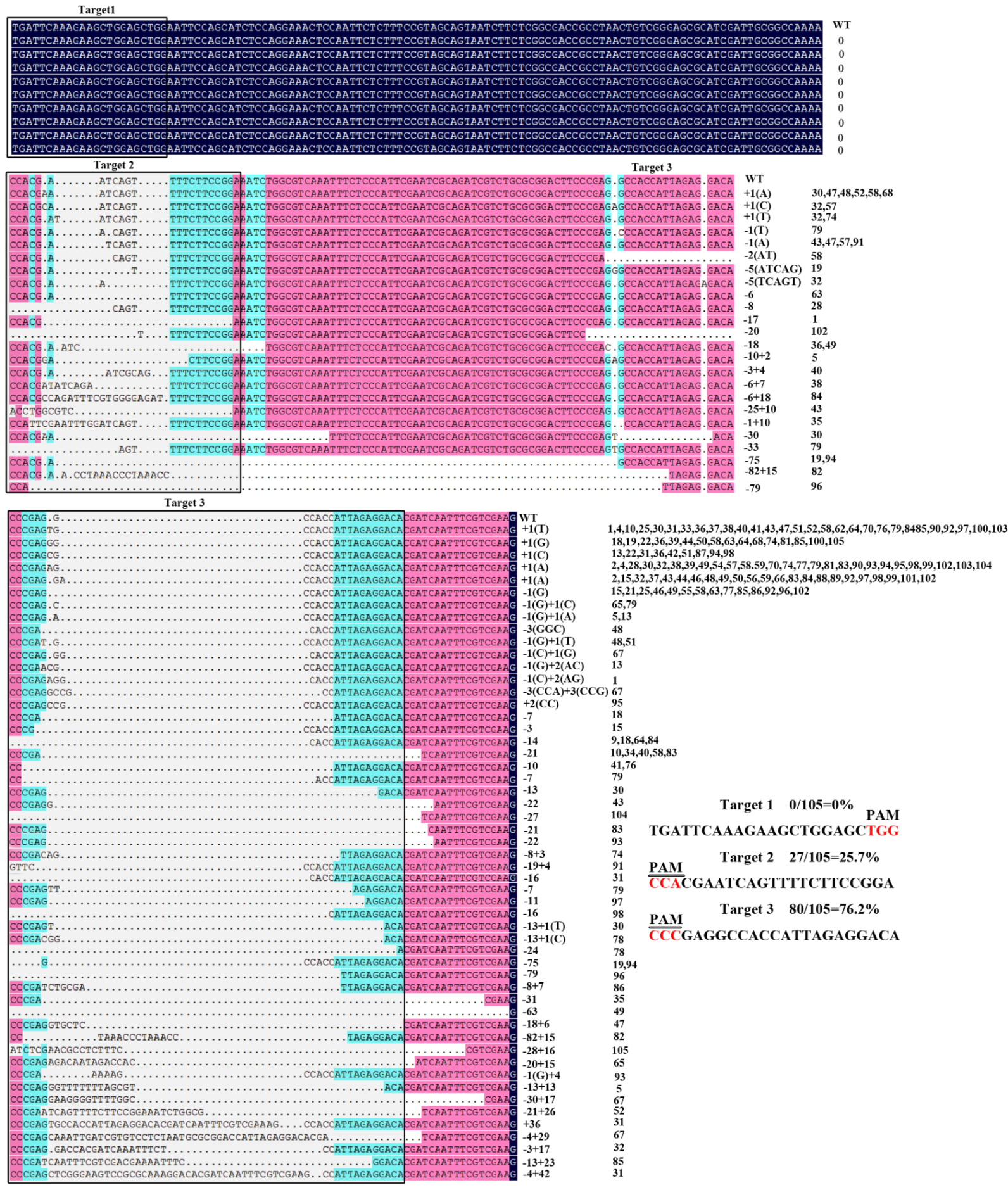


**Supplementary Figure S5.** Different types of mutations detected in the transgenic duckweed after CRISPR/Cas9-mediated gene editing. The plant numbers are shown in black.


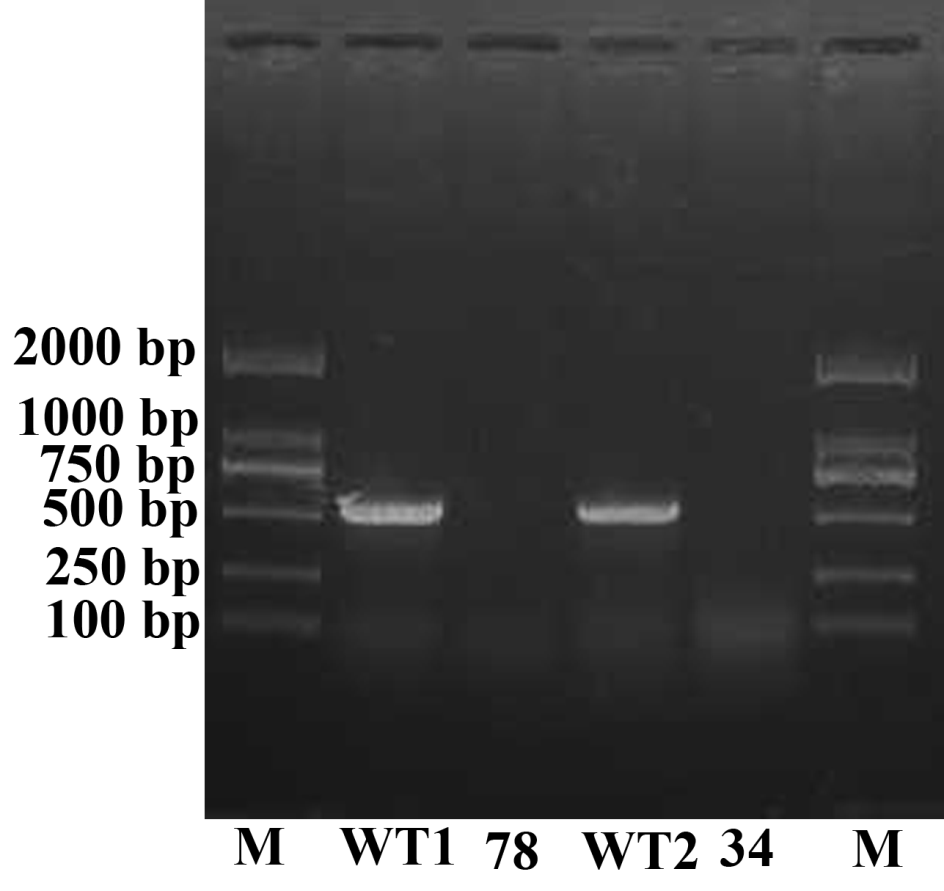


**Supplementary Figure S6.** Examine presence/absence of WT allele containing cells in two biallelic transformants. Amplifications were carried out with WT allele specific primers. Two mutants (ID: 78 and 34) that has large deletions were used. Lanes: M, molecular weight marker DL2000 (Takara); WT1 and WT2, wild-type plant DNA amplified with two sets of WT allele specific primers; #78 and #34, biallelic mutants.

**Supplementary Table S1.** Callus induction rate among different duckweed clones.

| Species | Clone | Place of collection | Latitude and longitude | Callus induction (mean±SD, in %) | |
| --- | --- | --- | --- | --- | --- |
| *Lemna aequinoctialis* | 6002 | Qingdao Shandong Province | 120°36’N, 36°57’E | | 98±4 |
| *Lemna aequinoctialis* | 6098 | Jining Shandong Province | 116°47’N, 34°98’E | | 96.5±3 |
| *Lemna aequinoctialis* | 6094 | Heyuan Guangdong Province | 114°91’N, 24°46’E | | 95.2±3 |
| *Lemna minor* | 487 | Waksman Collection | - | | 95.2±3 |
| *Lemna aequinoctialis* | 6095 | Xian Shanxi Province | 108°93’N, 34°41’E | | 94.5±4 |
| *Lemna aequinoctialis* | 6096 | Luoyang Henan Province | 112°48’N, 34°16’E | | 93.4±5 |
| *Lemna aequinoctialis* | 6097 | Qingdao Shandong Province | 120°41’N, 36°16’E | | 90.0±4 |
| *Lemna aequinoctialis* | 6003 | Qingdao Shandong Province | 120°41’N, 36°16’E | | 80 |
| *Lemna aequinoctialis* | 6007 | Qingdao Shandong Province | 120°42’N, 36°16’E | | 25 |
| *Lemna aequinoctialis* | 6037 | Jiaonan Shandong Province | 119°95’N, 35°88’E | | 25 |
| *Lemna aequinoctialis* | 6027 | Xianyang Shanxi Province | 107°72’N, 35°20’E | | 10 |
| *Lemna aequinoctialis* | 6004 | Hefei Anhui Province | 117°85’N, 31°60’E | | 10 |
| *Lemna aequinoctialis* | 6000 | Lixian Hunan Province | 111°77’N, 29°62’E | | 0 |
| *Lemna aequinoctialis* | 6005 | Lixian Hunan Province | 112°30’N, 29°41’E | | 0 |
| *Lemna aequinoctialis* | 6006 | Wuhu Anhui Province | 118°40’N, 31°35’E | | 0 |
| *Lemna aequinoctialis* | 6008 | Changde Hunan Province | 111°72’N, 29°05’E | | 0 |
| *Lemna aequinoctialis* | 6009 | Jinan Shandong Province | 116°97’N, 36°70’E | | 0 |
| *Lemna aequinoctialis* | 6010 | Jinggangshan Jiangxi Province | 114°17’N, 26°59’E | | 0 |
| *Lemna aequinoctialis* | 6011 | Qingdao Shandong Province | 120°44’N, 36°27’E | | 0 |
| *Lemna aequinoctialis* | 6012 | Jinggangshan Jiangxi Province | 114°17’N, 26°59’E | | 0 |
| *Lemna aequinoctialis* | 6013 | Chengdu Sichuan Province | 104°08’N, 30°65’E | | 0 |
| *Lemna aequinoctialis* | 6014 | Anyang Henan Province | 112°07’N, 29°42’E | | 0 |
| *Lemna aequinoctialis* | 6015 | Xingcheng Liaoning Province | 120°73’N, 40°63’E | | 0 |
| *Lemna aequinoctialis* | 6016 | Anhong Hunan Province | 112°21’N, 29°35’E | | 0 |
| *Lemna aequinoctialis* | 6017 | Qingdao Shandong Province | 120°37’N, 36°16’E | | 0 |
| *Lemna aequinoctialis* | 6018 | Qingdao Shandong Province | 112°44’N, 36°17’E | | 0 |
| *Lemna aequinoctialis* | 6019 | Qingdao Shandong Province | 112°44’N, 36°27’E | | 0 |
| *Lemna aequinoctialis* | 6020 | Taiyuan Shanxi Province | 112°56’N, 37°89’E | | 0 |
| *Lemna aequinoctialis* | 6021 | Nanchang Jiangxi Province | 115°84’N, 28°71’E | | 0 |
| *Lemna aequinoctialis* | 6022 | Sanya Hainan Province | 109°53’N, 18°25’E | | 0 |
| *Lemna aequinoctialis* | 6023 | Lianyugang Jiangsu Province | 119°17’N, 34°62’E | | 0 |
| *Lemna aequinoctialis* | 6024 | Wenzhou Zhejiang Province | 120°64’N, 27°99’E | | 0 |
| *Lemna aequinoctialis* | 6025 | Anzao Hunan Province | 112°19’N, 29°60’E | | 0 |
| *Lemna aequinoctialis* | 6026 | Anhong Hunan Province | 107°72’N, 29°34’E | | 0 |
| *Lemna aequinoctialis* | 6028 | Xianyang Shanxi Province | 107°72’N, 35°20’E | | 0 |
| *Lemna aequinoctialis* | 6029 | Jiaonan Shandong Province | 119°79’N, 35°68’E | | 0 |
| *Lemna aequinoctialis* | 6030 | Jiaonan Shandong Province | 119°79’N, 35°68’E | | 0 |
| *Lemna aequinoctialis* | 6031 | Changsha Hunan Province | 112°95’N, 28°21’E | | 0 |
| *Lemna aequinoctialis* | 6032 | Kunming Yunnan Province | 102°77’N, 24°81’E | | 0 |
| *Lemna aequinoctialis* | 6033 | Qingdao Shandong Province | 120°40’N, 36°16’E | | 0 |
| *Lemna aequinoctialis* | 6034 | Hefei Anhui Province | 117°85’N, 31°60’E | | 0 |
| *Lemna aequinoctialis* | 6035 | Anzhang Hunan Province | 112°18’N, 29°47’E | | 0 |
| *Lemna aequinoctialis* | 6036 | Fuqing Fujian Province | 119°39’N, 25°72’E | | 0 |
| *Lemna aequinoctialis* | 6038 | Changde Hunan Province | 111°71’N, 29°05’E | | 0 |
| *Lemna aequinoctialis* | 6039 | Shanghai City | 121°41’N, 31°23’E | | 0 |
| *Lemna aequinoctialis* | 6040 | Binzhou Shandong Province | 118°01’N, 37°42’E | | 0 |
| *Lemna aequinoctialis* | 6041 | Weifang Shandong Province | 119°41’N, 36°87’E | | 0 |
| *Lemna aequinoctialis* | 6042 | Honghe Yunnan Province | 102°73’N, 23°15’E | | 0 |
| *Lemna aequinoctialis* | 6043 | Xuchang Henan Province | 113°85’N, 34°05’E | | 0 |
| *Lemna aequinoctialis* | 6090 | Taian Shandong Provinve | 117°20’N, 36°07’E | | 0 |
| *Lemna aequinoctialis* | 6091 | Haerbin Heilongjiang Province | 126°70’N, 45°78’E | | 0 |
| *Lemna aequinoctialis* | 6092 | Weifang Shandong Province | 119°41’N, 36°87’E | | 0 |
| *Lemna aequinoctialis* | 6093 | Weihai Shandong Province | 122°08’N, 37°51’E | | 0 |
| *Lemna aequinoctialis* | 6099 | Tianjin City | 117°21’N, 39°09’E | | 0 |
| *Lemna aequinoctialis* | 6100 | Beiijing City | 116°49’N, 39°95’E | | 0 |
| *Lemna aequinoctialis* | 6101 | Anzhao Hunan Province | 112°19’N, 29°60’E | | 0 |
| *Lemna aequinoctialis* | 6102 | Huludao Liaoning Province | 120°85’N, 40°72’E | | 0 |
| *Lemna minor* | 6044 | Lianyungang Jiangsu Province | 119°17’N, 34°62’E | | 0 |
| *Landoltia punctata* | 6001 | Chongqing City | 106°52’N, 29°50’E | | 0 |
| *Landoltia punctata* | 6045 | Wuxi Jiangsu Province | 120°32’N, 31°50’E | | 0 |
| *Landoltia punctata* | 6046 | Chengdu Sichuan Province | 104°08’N, 30°65’E | | 0 |
| *Landoltia punctata* | 6047 | Qingdao Shandong Province | 120°49’N, 36°15’E | | 0 |
| *Landoltia punctata* | 6048 | Lixian Hunan Province | 111°78’N, 29°63’E | | 0 |
| *Landoltia punctata* | 6049 | Anhong Hunan Province | 112°21’N, 29°35’E | | 0 |
| *Landoltia punctata* | 6050 | Kunming Yunnan Province | 102°77’N, 240°81’E | | 0 |
| *Landoltia punctata* | 6051 | Anxiang Hunna Province | 112°19’N, 29°60’E | | 0 |
| *Landoltia punctata* | 6052 | Anhong Hunan Province | 112°21’N, 29°34’E | | 0 |
| *Landoltia punctata* | 6053 | Xianyang Shanxi Province | 107°78’N, 35°19’E | | 0 |
| *Landoltia punctata* | 6054 | Jiaonan Shandong Province | 119°80’N, 35°68’E | | 0 |
| *Landoltia punctata* | 6055 | Qingdao Shandong Province | 120°50’N, 36°17’E | | 0 |
| *Landoltia punctata* | 6056 | Xianyang Shanxi Province | 107°76’N, 35°21’E | | 0 |
| *Landoltia punctata* | 6057 | Qingdao Shandong Province | 120°44’N, 36°27’E | | 0 |
| *Landoltia punctata* | 6058 | Anxiang Hunan Province | 112°18’N, 29°49’E | | 0 |
| *Landoltia punctata* | 6059 | Shaoxing Zhejiang Province | 120°58’N, 30°03’E | | 0 |
| *Landoltia punctata* | 6060 | Qianan Hebei Province | 118°69’N, 39°98’E | | 0 |
| *Landoltia punctata* | 6061 | Yichang Hubei Province | 111°32’N, 30°74’E | | 0 |
| *Landoltia punctata* | 6062 | Chongqing City | 106°52’N, 29°50’E | | 0 |
| *Landoltia punctata* | 6063 | Lanzhou Gansu Province | 104°12’N, 35°87’E | | 0 |
| *Landoltia punctata* | 6064 | Nanning Guangxi Province | 108°36’N, 22°81’E | | 0 |
| *Landoltia punctata* | 6065 | Kunming Yunnan Province | 102°77’N, 24°1’E | | 0 |
| *Landoltia punctata* | 6066 | Taian Shandong Province | 117°12’N, 36°21’E | | 0 |
| *Landoltia punctata* | 6067 | Kunming Yunnan Province | 102°77’N, 24°81’E | | 0 |
| *Spirodela polyrhiza* | 6068 | Anzhang Hunan Province | 112°18’N, 29°48’E | | 0 |
| *Spirodela polyrhiza* | 6069 | Lianyungang Jiangsu Province | 119°16’N, 34°62’E | | 0 |
| *Spirodela polyrhiza* | 6070 | Anxiang Hunan Province | 112°29’N, 29°41’E | | 0 |
| *Spirodela polyrhiza* | 6071 | Taian Shandong Province | 117°19’N, 36°07’E | | 0 |
| *Spirodela polyrhiza* | 6072 | Qingdao Shandong Province | 120°42’N, 36°16’E | | 0 |
| *Spirodela polyrhiza* | 6073 | Qingdao Shandong Province | 119°79’N, 35°68’E | | 0 |
| *Spirodela polyrhiza* | 6074 | Anhong Hunan Province | 112°21’N, 29°35’E | | 0 |
| *Spirodela polyrhiza* | 6075 | Liupanshui Guizhou Province | 104°83’N, 26°61’E | | 0 |
| *Spirodela polyrhiza* | 6076 | Qingdao Shandong Province | 120°50’N, 36°16’E | | 0 |
| *Spirodela polyrhiza* | 6077 | Tangshan Tianjin Province | 118°92’N, 39°42’E | | 0 |
| *Spirodela polyrhiza* | 6078 | Lixian Hunan Province | 111°78’N, 29°63’E | | 0 |
| *Spirodela polyrhiza* | 6079 | Jining Shandong Province | 117°00’N, 35°59’E | | 0 |
| *Spirodela polyrhiza* | 6080 | Jinan Shandong Province | 116°97’N, 36°70’E | | 0 |
| *Spirodela polyrhiza* | 6081 | Xuchang Henan Province | 113°84’N, 34°04’E | | 0 |
| *Spirodela polyrhiza* | 6082 | Anxiang Hunan Province | 112°29’N, 29°41’E | | 0 |
| *Spirodela polyrhiza* | 6083 | Jining Shandong Province | 116°98’N, 34°94’E | | 0 |
| *Spirodela polyrhiza* | 6084 | Changsha Hunan Province | 112°95’N, 28°22’E | | 0 |
| *Spirodela polyrhiza* | 6085 | Xingcheng Liaoning Province | 120°72’N, 40°62’E | | 0 |
| *Spirodela polyrhiza* | 6086 | Anhong Hunan Province | 112°21’N, 29°35’E | | 0 |
| *Spirodela polyrhiza* | 6087 | Qingdao Shandong Province | 120°26’N, 36°21’E | | 0 |
| *Spirodela polyrhiza* | 6088 | Jinan Shandong Province | 116°95’N, 36°67’E | | 0 |
| *Spirodela polyrhiza* | 6089 | Nanchang Jiangxi Province | 115°84’N, 28°71’E | | 0 |
| *Spirodela polyrhiza* | 7498 | North Carolina | -78°53’S, 35°59’E | | 0 |

**Supplementary Table S2.** Genetic transformation methods that were used in duckweeds.

| Species | Explant | | Co-cultivation Medium | Induction Medium | *Agrobacterium* | | | | | | Infection method | Regeneration medium | | | Efficiency | | | | | | Reference | | | |
| --- | --- | --- | --- | --- | --- | --- | --- | --- | --- | --- | --- | --- | --- | --- | --- | --- | --- | --- | --- | --- | --- | --- | --- | --- |
| *Lemna aequinoctialis*  6002 | | Callus | SH medium with 10 g/L sucrose | MS medium containing 30 g/L sucrose, 4.52 μM 2,4-D, 0.45 μM TDZ and 7.9 g/L bacteriological agar | | EHA105 | | Vacuum infiltration for 10 min, sonication for 5 min, vacuum infiltration for 10 min and shaking for 30 min | | | | | | B5 basal medium supplemented with 4.65 μM kinetin, 2.57 μM IAA, 1% sucrose, 9.48 μM hygromycin, 600 μM timentin | | | | 94% | | This study | | | |  |
| *Lemna gibba* G3  *Lemna*  *minor* 8627 | | Callus | SH medium with 10 g/L sucrose | MS medium containing 30 g/L sucrose, 5 μM 2,4-D, 0.5 μM TDZ and 5 g/L bacteriological agar | | | GV3101 | | Submerged for 5 min | | | | 10 g/L sucrose, 200 mg/L carbenicillin, 500 mg/L cefotaxamin, 10 mg/L PPT, 5 g/L bacteriological agar | | | | | 59% | | Cantó-Pastor  *et al.*,  2015 | | | |  |
| *Lemna minor* | | Callus | Hoagland’s medium containing 10 μM 6-BA | B5 medium containing 1% sucrose, 5.0 μM 2,4-D, 50.0 μM 2-iP or 50.0 μM 2,4-D 5.0 μM TDZ and 0.35% phytagel | | | EHA105 | | Prewashed with 0.2% Tween 20, shaking at 80 rpm for 60 min | | | B5 medium containing 1% sucrose, 5.0 μM kinetin, 25.0 μM IAA, 125 mg/L kanamycin and 500 mg/L cefotaxime | | | | | | 10% | | Chhabra *et*  *al.*, 2011 | | | |  |
| *Lemna minor* ZH0055 | | Callus | Hoagland liquid medium containing 1.5% sucrose | MS medium containing 30 g/L sucrose, 10 mg/L 2,4-D, 0.5 mg/L 2-iP and 3 g/L gelrite | | | EHA105 | | Submerged and vacuum infiltrated for 10 min | | | MS medium containing 30 g/L sucrose, 10 mg/L 2,4-D, 0.5 mg/L 2-iP, 3 g/L gelrite, 100 mg/L timentin, 100 mg/L paromomycin, 20 mg/L G418 | | | | | | 80% | | Yang *et al.*,  2018a | | | |  |
| *Lemna minor* ZH0055, 6580, 6591 | | Frond | Hoagland liquid medium containing 1.5% sucrose | - | | | EHA105 | | Vacuum infiltrated for 10 min | | | MS medium containing 30 g/L sucrose, 10 mg/L 2,4-D, 0.5 mg/L 2-iP, 3 g/L gelrite, 100 mg/L timentin, 100 mg/L paromomycin and 20 mg/L G418 | | | | | | 40% | | Yang *et al.*,  2018a | | | |  |
| *Lemna*  *minor 8627, 8744 Lemna gibba G3* | | Callus | Liquid SH medium with 10 g/L sucrose | MS medium containing, 30 g/L sucrose, 4 g/L Difco Bacto-agar, 1.5 g/L gelrite, 10 mM NAA and 0.5 mM TDZ (*L. gibba* G3) or 5 mM 2,4-D and 0.5 mM TDZ (*L. minor*) | | | C58-z707 | | | Submerged for 3-5 min | | MS basal medium containing 30 g/1 sucrose, 1 mM 2,4-D, 2 mM 6-BA, 4 g/L Difco Bacto-agar, 1.5 g/L gelrite, 10 mg/L kanamycin and 500 mg/L cefotaxime | | | | | | - | | Yamamoto *et*  *al.*, 2001;  Sun *et al.*,  2007 | | |  |  |
| *Lemna minor* | | Frond | - | Halfstrength MS medium supplemented with 1 mg/L 6-BA, 0.4 mg/L thiamine HCl, 100 mg/L myo-inositol, 15 g/L sucrose and 4 g/L gelrite | | | EHA105 | | Submerged for 30 min | | | Halfstrength MS medium supplemented with 1 mg/L 6-BA, 0.4 mg/L thiamine HCl, 100 mg/L myo-inositol, 15 g/L sucrose and 4 g/L gelrite, 300 mg/L cefotaxime and 200 mg/L kanamycin | | | | | | - | | Ko *et al.*,  2011 | | |  |  |
| *Lemna minor* *Lemna turionifera* 5511 | | Callus | - | B5 medium containing 1.5 % sucrose, 15 mg/L dicamba, 3.5 mg/L 2,4-D and 1 mg/L 6-BA | | | EHA105 | | Prewashed with 0.2% Tween 20 and shaking at 80 rpm for 60 min | | | B5 medium containing 1.5% sucrose, 1 mM L-Ser, 1 mg/L hygromycin | | | | | | - | | Yang *et al.*,  2013, 2017 | | |  |  |
| *Lemna minor* | | Callus | Liquid hormone-free MS medium containing 2.0 % sucrose | MS containing 3.0% sucrose, 0.4% agar, 0.15% gelrite and 1.0 mg/L TDZ | | | CBE21 | | Incubated for  30 min | | | MS containing 3.0% sucrose, 0.4% agar, 0.15% gelrite, 2.0 mg/L benzylaminopurine, 0.1 mg/L IAA, 200 mg/L cefotaxime and 35 mg/L kanamycin | | | | | 82.5% | | Firsov *et al.*, 2015, 2018 | | |  |  |  |
| *Spirodela oligorrhiza* SP^*^ | | Callus | Half-strength Hutner’s medium | McCown Woody Plant medium containing 1.5% galactose, 50 mg/L dicamba and 2 mg/L 6-BA | | | EHA105 | | Wounded by DNA-free tungsten particles, submerged and vacuum infiltrated for 5 min | | | Liquid SP medium containing 1% sucrose, 200 mg/L carbenicillin and 2–5 mg/L kanamycin | | | | | 5% | | Vunsh *et al.*, 2007; Rival *et al.*, 2008 | | |  |  |  |
| *Spirodela polyrhiza* 5543 | | Callus | Half-strength MS medium containing 1% sucrose | Half-strength MS medium containing 1% sucrose, 22.62 μM 2,4-D and 8.88 μM 6-BA | | LBA4404 | | | Shaking for 20 min at 80–100 rpm, vacuum infiltrated for 10 min | | | Half-strength MS medium containing 1% sucrose, 0.6% agar, 9.99 mM (NH_4_)_2_SO_4_, 22.62 μM 2,4-D, 8.88 μM 6-BA, 0.63 mM cefotaxime and 96.25 μM G418 | | | | | 13% | | Yang *et al.*, 2018b | | |  |  |  |
| *Wolffia arrhiza* 5564 | | Cluster | SH medium containing 1% mannitol, 1% sorbitol, 2% glucose, 5.0 mg/L 2,4-D and 0.5 mg/L 6-BA | SH medium containing 1 % mannitol, 1 % sorbitol, 2 % glucose, 2.0 mg/L 2,4-D, 2.0 mg/L 6-BA | | | EHA105 | | Shaking for 30 min | | | SH media containing 1% mannitol, 1% sorbitol, 2% glucose and 5.0 mg/L hygromycin | | | | | 0.4% | | Khvatkov *et al.*, 2015, 2018 | | |  |  |  |
| *Wolffia globosa* 5563 | | Cluster | SH medium containing 5.0 mg/L 2,4-D and 0.5 mg/L 6-BA | Liquid SH containing 1% sorbitol, 5% sucrose and 10 μM AS | | | EHA105 | | Shaking with glass beads at 180 rpm for 30 min | | | SH media containing 2% sucrose, 150 mg/L cefotoxime and 40 mg/L G418 | | | | 0.14% | | | Heenatigala *et al.*, 2018 | | |  |  |  |

^*^*Spirodela oligorrhiza* was grouped with *Spirodela punctata* as discussed by Landolt, which was subsequently changed to a distinct genus and became *Landoltia punctata*. 2,4-D, 2,4-Dichlorophenoxyacetic acid; 2-iP, N6-(delta 2-Isopentenyl)-adenine; 6-BA, 6-benzyladenine; IAA, Indole-3-acetic acid; NAA, 1-naphthaleneacetic acid; PPT, phosphinothricin; TDZ, Thidiazuron.

**Supplementary Table S3.** Chlorophyll *a*, chlorophyll *b* and total carotenoids content of transgenic lines. Line 58, 59 and 62 are chimeric mutant lines; line 78, 82 and 103 are biallelic mutant lines. Pigment is the summation of chlorophyll and carotenoids. Values are mean ± SD (n=3). The results are expressed as mg.gDW^-1^. DW: dry weight.

| Different transgene line | Chlorophyll *a* content | Chlorophyll *b* content | Total carotenoids content | Pigment content |
| --- | --- | --- | --- | --- |
| WT | 8.70±0.24 | 3.56±0.018 | 2.18±0.035 | 14.44±0.45 |
| 58 | 6.05±0.11 | 2.42±0.039 | 1.54±0.04 | 10.01±0.15 |
| 59 | 7.53±0.78 | 3.44±0.17 | 1.81±0.17 | 12.79±0.78 |
| 62 | 5.50±0.33 | 2.35±0.16 | 1.31±0.065 | 9.16±0.55 |
| 78 | 2.15±0.034 | 0.94±0.027 | 0.48±0.0073 | 3.57±0.068 |
| 82 | 1.59±0.084 | 0.73±0.038 | 0.33±0.021 | 2.64±0.13 |
| 103 | 0.21±0.041 | 0.16±0.019 | 0.052±0.016 | 0.43±0.076 |

**Supplementary Table S4.** Primers used in this study.

| **Primer name** | **Primer sequence** | **Aims** |
| --- | --- | --- |
| *LaPDS*-F  *LaPDS*-R | 5´-ATGGGATACACCGGCTCCGTCTCCG-3´  5´-TAGGCCACGGCTGGTTCGACCTTA-3´ | Amplification of *LaPDS* |
| *LaPDS*-F1: *LaPDS*-R1: | 5´-CGCCGCCGGCTCCTTCACCCTT-3´  5´-CCTGCTCCCGCAATGACGACTT-3´ | Identification of mutation |
| *PDS*-U3F  *PDS*-U3R | 5´-GGCATGATTCAAAGAAGCTGGAGC-3´  5´-AAACGCTCCAGCTTCTTTGAATCA-3´ | Target1 adapter |
| \| *PDS*-U6aF \| \| --- \| \| *PDS*-U6aR \| | 5´-GCCGTCCGGAAGAAAACTGATTCG-3´  5´-AAACCGAATCAGTTTTCTTCCGGA-3´ | Target2 adapter |
| \| *PDS*-U6bF \| \| --- \| \| *PDS*-U6bR \| | 5´-GTTGTGTCCTCTAATGGTGGCCTC-3´  5´-AAACGAGGCCACCATTAGAGGACA-3´ | Target3 adapter |
| U-F  gRNA-R | 5´-CTCCGTTTTACCTGTGGAATCG-3´  5´-CGGAGGAAAATTCCATCCAC-3´ | Cloning of sgRNA expression cassette |
| \| B1’ \| \| --- \| \| B2 \| | 5´-TTCAGAGGTCTCTCTCGCACTGGAATCGGCAGCAAAGG-3´  5´-AGCGTGGGTCTCGTCAGGGTCCATCCACTCCAAGCTC-3´ | Amplification of U3-target1-gRNA |
| \| B2’ \| \| --- \| \| B3 \| | 5´-TTCAGAGGTCTCTCTGACACTGGAATCGGCAGCAAAGG-3´  5´-AGCGTGGGTCTCGTCTTGGTCCATCCACTCCAAGCTC-3´ | Amplification of U6a-target2-gRNA |
| \| B3’ \| \| --- \| \| BL \| | 5´-TTCAGAGGTCTCTAAGACACTGGAATCGGCAGCAAAGG-3´  5´-AGCGTGGGTCTCGACCGGGTCCATCCACTCCAAGCTC-3´ | Amplification of U6b-target3-gRNA |
| FP6  FP7  FP8  FSP1  FSP2 | 5´-GTAATACGACTCACTATAGGGCACGCGTGGTNGACGASWGA-3´ 5´-GTAATACGACTCACTATAGGGCACGCGTGGTNGACGASWGANAWGAA-3´  5´-GTAATACGACTCACTATAGGGCACGCGTGGTGTNCGASWCANAWGTT-3´  5´-GTAATACGACTCACTATAGGGC-3´  5´-ACTATAGGGCACGCGTGGT-3´ | Tail-PCR degenerate primers |
| RB-0b  RB-1b  RB-2b | 5´-CGTGACTGGGAAAACCCTGGCGTT-3´  5´-CAACTTAATCGCCTTGCAGCACATC-3´  5´-CCCAACAGTTGCGCAGCCTGAATG-3´ | Tail-PCR specific primers |
| 34-F 5´-GGCCACCATTAGAGGACACG-3´  34-R 5´-CTCCTTCCATTGCAGTCGGT-3´  78-F 5´-ACTTCCCGAGGCCACCATTAG-3´  78-R 5´-CTCCTTCCATTGCAGTCGGT-3´ | | Validate WT cells absence in #34 and #78 biallelic mutants |
| *EHA105-crds*-F 5´-TGTATTTCAGTGCTGAAGGTG-3´  *EHA105-crds-*R 5´-AAATAGAATTGAGGCGTCTGG-3´ | | Examine *Agrobacterium* presence/absence in the regenerated tissue |
